# Hyperthyroidism in thyroid carcinoma originating in *struma ovarii*

**DOI:** 10.1530/EDM-24-0082

**Published:** 2024-10-03

**Authors:** Juliana Bezerra Mesquita, Rosa Paula M Biscolla

**Affiliations:** 1 Liga Norte Riograndense Contra o Cancer, Natal, Brazil; 2 Thyroid Diseases Center and Laboratory of Molecular and Translational Endocrinology, Division of Endocrinology, Department of Medicine, Escola Paulista de Medicina, Universidade Federal de São Paulo, São Paulo, Brazil

**Keywords:** thyroid carcinoma, Struma ovarii, hyperthyroidism, thyroid ectopic cancer

## Abstract

**Summary:**

Thyroid carcinoma originating in *Struma Ovarii* (SO) is a rare thyroid ectopic cancer that accounts for 0.01% of all ovarian malignancies and is associated with hyperthyroidism in less than 15% of cases. In a 44-year-old patient with pelvic pain, the CT scan revealed a solid-cystic formation in the ovarium. A left oophorectomy was performed and showed a borderline serous tumor and papillary thyroid carcinoma (‘thyroid carcinoma originating in *Struma Ovarii*’) measuring 10 cm. Thyroid function was assessed, and hyperthyroidism was diagnosed. Surgical complementation and a pelvic re-approach were performed. The histological findings showed a papillary thyroid carcinoma in the uterine serosa and the right adnexa. Thyroid function was re-evaluated, and despite normal thyroid function, the TRAb test remained positive. The patient underwent total thyroidectomy and radioiodine therapy (RIT), after which the TRAb test became negative. During 3 years of follow-up, no evidence of tumor was observed. In our case of thyroid carcinoma originating in SO, hyperthyroidism was treated with ovarian surgery, total thyroidectomy, and RIT. It is worth noting that thyroid function was normalized after ovarian surgery, but the TRAb test only became negative after total thyroidectomy. We hope to draw attention to the importance of evaluating thyroid function in patients with SO and treating high-risk SO patients with RIT after total thyroidectomy to achieve disease remission.

**Learning points:**

- *Struma ovarii* can cause hyperthyroidism.
- Thyroid carcinoma can originate in *Struma Ovarii.*
- Differentiated thyroid carcinoma and hyperthyroidism originating in *Struma Ovarii* are rare conditions.

## Background

Ovarian teratomas are germ-cell tumors that account for approximately 20% of all ovarian tumors (1). Up to 20% of ovarian teratomas contain thyroid tissue, but only 5% of these teratomas contain more than 50% thyroid tissue and are termed *Struma Ovarii* (SO) (1). Ninety-five percent of SO cases are benign, and 5–20% of SO cases are associated with hyperthyroidism (2). Less than 5% of SO cases progress to malignancy (2). In this article, we report the diagnosis and treatment of a patient with thyroid carcinoma originating in SO and hyperthyroidism.

## Case presentation

A 44-year-old patient presented with pelvic pain. She had regular menstrual cycles and four previous pregnancies. Pelvic ultrasonography (US) revealed two heterogeneous, septate, and regular images with anechoic textures measuring 3.4 cm and 4.0 cm in the left adnexa. A computed tomography (CT) scan showed a large heterogeneous, solid-cystic formation with thick septa, calcifications, and a fat component, measuring 9.0 × 3.6 cm. CA125 and CEA measurements were normal.

**Figure 1:**
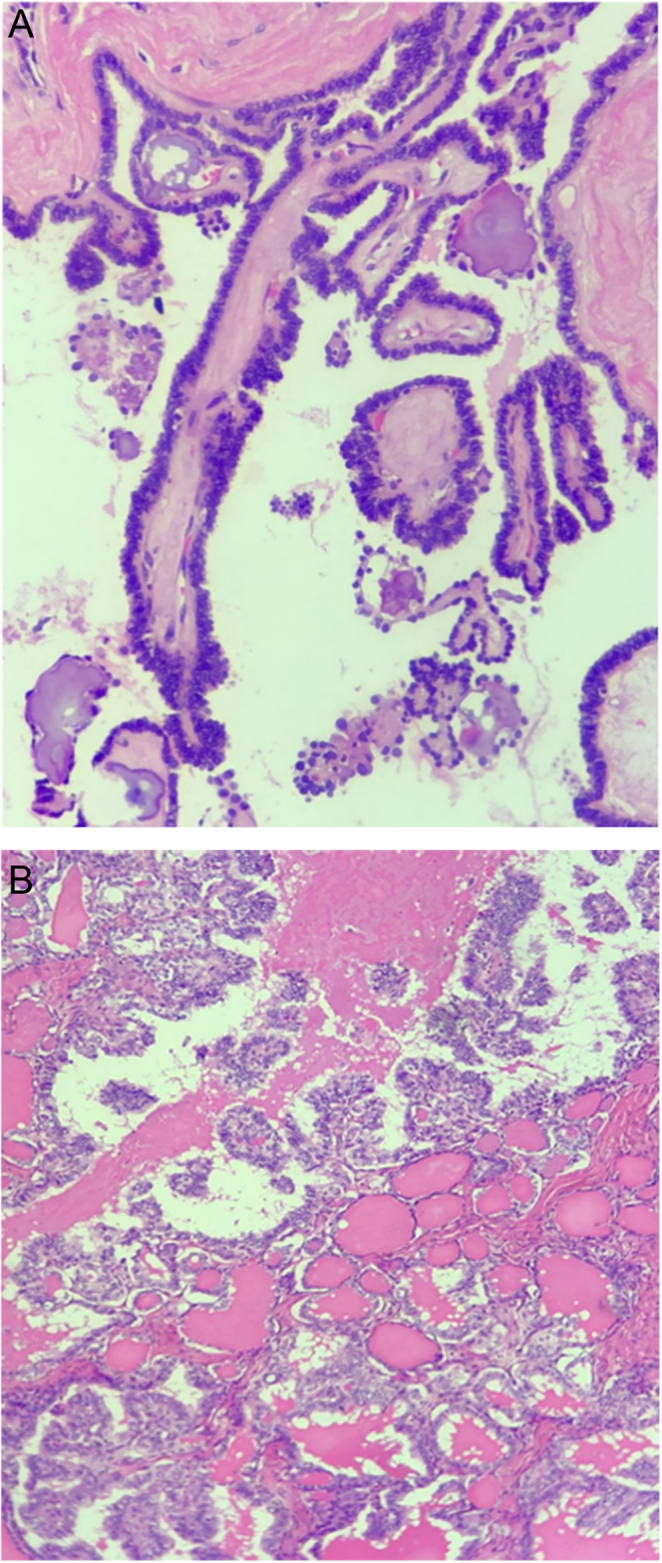
(A) Optical microscopy. Microscopic histological findings of the cystic areas of the ovarian lesion, lined by simple cubic epithelium, serous, with mild nuclear atypia and formation of branched papillae, compatible with a borderline serous tumor of the ovary. Staining: Hematoxylin-eosin. Magnification: 400x. (B) Optical microscopy. Microscopic histological findings of the solid areas in the ovarian lesion indicating the presence of benign thyroid tissue consisting of dilated follicles lined with flat cells containing an eosinophilic substance (colloid) inside the central region. Also, note epithelial proliferation with an architectural pattern consisting of papillae and lined by cubic and cylindrical cells, with nuclear alterations such as chromatin whitening and nuclear grooves, compatible with papillary thyroid carcinoma. Staining: Hematoxylin-eosin. Magnification: 100x.

A left oophorectomy was performed, and the pathologic report showed a borderline serous tumor and a papillary thyroid carcinoma (‘thyroid carcinoma originating in Struma Ovarii’) measuring 10 cm × 9.5 and no vascular/perineural invasion or surface ovarian involvement were observed (pT1apNx according to the AJCC 8th edition) (Fig. 1A and B).

After surgery, even though the patient was asymptomatic, thyroid function was evaluated: TSH: < 0.08 mU/L (RR: 0.45–4.0 mUI/L); fT4: 1.9 ng/dL (RR: 0.7–1.8); thyroglobulin (Tg): 54.5 ng/mL (RR: 1.4–78 ng/mL); positive thyroglobulin antibodies (TgAb) and TRAb: 2.28 UI/L (RR: <1.75 UI/L) indicating hyperthyroidism and the presence of an autoimmune disease. Thyroid US showed a normal volume of 12.5 cm³, and heterogeneous echotexture, without any nodules. Methimazole was started to control the hyperthyroidism.

Surgical complementation with a pelvic re-approach was performed: hysterectomy, right oophorectomy, bilateral salpingectomy, lymphadenectomy, and omentectomy, according to the institution’s protocol. The histological findings showed a papillary thyroid carcinoma in the uterine serosa (1 mm), a metastatic papillary carcinoma in the right adnexa (5 mm), and ovarian surface involvement. After surgery, methimazole was withdrawn, thyroid function normalized, and thyroid scintigraphy showed normal uptake, but the TRAb test and thyroglobulin antibodies remained positive.

Due to the characteristics of the SO (size, metastatic implants), a total thyroidectomy was performed, with the aim of administering adjuvant radioiodine therapy to the patient. The pathologic diagnosis was lymphocytic thyroiditis. After 20 days, the TRAb test turned negative. The patient was treated with 100 mCi (3700 MBq) of radioiodine [I]-131 therapy (RIT) and presented TSH 30 mUI/L, thyroglobulin 35.8 ng/mL, and positive thyroglobulin antibodies (TgAb). Whole-body scintigraphy (WBS) showed slight uptake in the anterior cervical region. Screening for oncological disease via chest, abdomen, and pelvis CTs showed no findings. Three years later, the patient remained asymptomatic and negative for all anatomical tumor evidence and tumor markers (thyroglobulin, TgAb, and CA125). As shown in Table 1, the thyroid function normalized and tumor markers became negative during follow-up.

**Table 1:** Thyroid function and thyroglobulin levels during follow up.

	TSH (µUI/mL)	fT4 (ng/dL)	T3 (ng/dL)	Tg (ng/mL)	TgAb (IU/mL)
After left oophorectomy	<0.08	1.9	187	54.5	331
After surgical complementation	0.04	1.2		63.3	348
Before total thyroidectomy	1.31	1.1		44.5	280
After total thyroidectomy	0.94	1.4		8.23	270
Immediately before RIT	30	–		35.8	136.9
Three years later	0.29	1.01		0.11	20
Reference range	0.4–4.3	0.7–1.8	70–204	1.4–78	<115

Tg, thyroglobulin; TgAb, thyroglobulin antibodies.

## Discussion

*Struma ovarii* is a rare ovarian tumor that affects mainly women between the ages of 40 and 60 years (3). It was first described in 1889 by Boettlin and later published by Gottschalk (3). In the majority of cases, SO is diagnosed after surgery for the investigation of an ovarian mass. The symptoms are nonspecific, such as abdominal pain, ascites, a pelvic mass, and abnormal vaginal bleeding (3). Ascites has been reported in 15–20% of cases, and it is not necessarily a sign of malignancy (4). Unilateral presentation is more frequent, occurring in 90% of affected patients (5). A total of 18.5% of patients with thyroid carcinoma originating in SO are asymptomatic (6). Pelvic US and magnetic resonance imaging show heterogeneous images with cystic areas in the ovary, and the measurement of CA125 may be normal (7).

The thyroid tissue in SO is chemically, pharmacologically, biologically, and microscopically identical to the thyroid gland (4). On neck US, thyroid volume is normal, but serum thyroglobulin is usually elevated.

In this article, we report a rare case of thyroid carcinoma originating in SO complicated with hyperthyroidism. Hyperthyroidism has been reported in 5–15% (8) of patients with SO and is more common in those with benign SO than in those with thyroid carcinoma originating in SO (6). The cause of hyperthyroidism in SO could be associated with the hyperfunctioning of SO tissue alone or associated with cervical goiter (Graves’ disease) or Graves’ disease with nonfunctioning SO (5).

However, hyperfunctioning SO thyroid tissue has no histopathological characteristic findings in Graves’ disease tissue (5), if hyperthyroidism is caused by concomitant hyperfunctioning SO and Graves’ disease, TRAb should be considered in thyrocyte growth (5). Mimura *et al.* speculated that the presence of TRAb could stimulate thyroid tumor tissue in patients with SO (5).

Our patient with SO presented with clinical hyperthyroidism, likely due to hyperfunctioning thyroid tumor tissue associated with Graves’ disease. Histological examination revealed lymphocytic thyroiditis, indicative of an autoimmune thyroid disease background. Although thyroid function tests normalized following SO removal, the TRAb test turned negative only after total thyroidectomy. This observation aligns with the literature, which suggests that serum TRAb levels can decrease rapidly within the first 3 months post-total thyroidectomy, primarily due to the removal of intrathyroidal B lymphocytes (9). Regarding thyroid cancer and SO, papillary carcinoma is the histological subtype that accounts for approximately 70% of thyroid carcinoma originating in SO cases (7).

There is no standard therapy recommended for thyroid carcinoma originating in SO. Management is based on case reports and the risk of disease. Yassa *et al.* suggested that patients with a tumor under 2 cm in size that is confined to the ovary and does not show any aggressive features should be classified as low risk, whereas patients who present with a tumor that is larger than 2 cm in size or shows extraovarian extension are considered high risk (10). In 2018, Lebreton *et al.* suggested that a papillary carcinoma that is intracapsulated, well-differentiated, and completely removed after surgery was low risk for SO malignancy, but a tumor that is 4–5 cm in size or shows intense mitotic activity, tumor necrosis, vascular or lymphatic embolization, or poor differentiation was high risk for malignancy (11). These classifications of low- and high-risk guide the choice of local/pelvic surgery (cystectomy, oophorectomy with unilateral salpingectomy, to hysterectomy and bilateral oophorectomy and salpingectomy) for low-risk patients and the choice of local/pelvic surgery, including total thyroidectomy and RIT, for high-risk patients (10, 11). The follow-up is based on the measurement of serum thyroglobulin and thyroid function 10 years posttreatment (12).

In our patient, the histological finding was a papillary carcinoma. She was classified as high risk because of the tumor size (10 cm) and multiple foci of the papillary thyroid carcinoma that were located in the uterus’ serosa and in the right adnexa and ovarian surface. She was treated with total thyroidectomy and RIT (100 mCi). After 3 years of follow-up, thyroglobulin and anti-thyroglobulin antibodies remained undetectable, and pelvic ultrasound showed no evidence of disease.

In conclusion, we present a rare case of thyroid carcinoma originating in SO and concurrent Graves’ disease with hyperthyroidism, which was successfully treated with ovarian surgery, total thyroidectomy, and RIT. We hope to draw attention to the importance of assessing thyroid function in patients with SO, and considering the co-existence of Graves’ disease in the treatment strategy to manage hyperthyroidism. Additionally, the treatment of high-risk SO patients with RIT after total thyroidectomy is essential for achieving complete disease remission.

## Declaration of interest

The authors declare that there is no conflict of interest that could be perceived as prejudicing the impartiality of the study reported.

## Funding

This work did not receive any specific grant from any funding agency in the public, commercial, or not-for-profit sector.

## Patient consent

Written consent has been obtained from the patient after full explanation of the purpose and nature of all procedures used. This work has been approved by the ethics and research committee at Liga Norte Riograndense Contra o Câncer (CAAE 37173620.3.0000.5293), Natal, RN, Brazil, and the patient completed the informed consent form.

## Author contribution statement

JBM: Conceptualization (lead); writing – original draft (lead); formal analysis (lead); writing – review and editing (equal). RPMB: Conceptualization (lead); writing – original draft (lead); formal analysis (lead); writing – review and editing (equal).

## Acknowledgement

We would like to acknowledge the pathologist Thyago Mascicano for having provided the images.

## References

[1] Tan A Stewart CJ Garrett KL Rye M & Cohen PA. Novel BRAF and KRAS mutations in papillary thyroid carcinoma arising in struma ovarii. Endocrine Pathology 2015 26 296–301. (10.1007/s12022-015-9394-3) 26362194

[2] Grandet PJ & Remi MH. Struma ovarii with hyperthyroidism. Clinical Nuclear Medicine 2000 25 763–765. (10.1097/00003072-200010000-00001) 11043711

[3] Yoo SC Chang KH Lyu MO Chang SJ Ryu HS & Kim HS. Clinical characteristics of struma ovarii. Journal of Gynecologic Oncology 2008 19 135–138. (10.3802/jgo.2008.19.2.135) 19471561 PMC2676458

[4] Plaut A. Ovarian struma: a morphologic, pharmacologic, and biologic examination. American Journal of Obstetrics and Gynecology 1933 25 351–359. (10.1016/s0002-9378(33)90239-2)

[5] Mimura Y Kishida M Masuyama H Suwaki N Kodama J Otsuka F Kataoka H Yamauchi T Ogura T Kudo T, *et al.* Coexistence of Graves’ disease and struma ovarii: case report and literature review. Endocrine Journal 2001 48 255–260. (10.1507/endocrj.48.255) 11456276

[6] Zhang R Tian X Luo Y Dong H Tian W Zhang Y Li D Sun H & Meng Z. Case report: recurrent malignant struma ovarii with hyperthyroidism and metastases, a rare case report and review of the literature. Pathology Oncology Research 2022 28 1610221. (10.3389/pore.2022.1610221) 35620742 PMC9127674

[7] Robboy SJ Shaco-Levy R Peng RY Snyder MJ Donahue J Bentley RC Bean S Krigman HR Roth LM & Young RH. Malignant struma ovarii: an analysis of 88 cases, including 27 with extraovarian spread. International Journal of Gynecological Pathology 2009 28 405–422. (10.1097/PGP.0b013e3181a27777) 19696610

[8] Marcus CC & Marcus SL. Struma ovarii. A report of 7 cases and a review of the subject. American Journal of Obstetrics and Gynecology 1961 81 752–762. (10.1016/S0002-9378(1533524-9) 13766579

[9] Yoshioka W Miyauchi A Ito M Kudo T Tamai H Nishihara E Kihara M Miya A & Amino N. Kinetic analyses of changes in serum TSH receptor antibody values after total thyroidectomy in patients with Graves’ disease. Endocrine Journal 2016 63 179–185. (10.1507/endocrj.EJ15-0492) 26632172

[10] Yassa L Sadow P & Marqusee E. Malignant struma ovarii. Nature Clinical Practice. Endocrinology and Metabolism 2008 4 469–472. (10.1038/ncpendmet0887) 18560398

[11] Lebreton C Al Ghuzlan A Floquet A Kind M Leboulleux S & Godbert Y. Cancer thyroïdien sur struma ovarii: généralités et principes de prise en charge. Bulletin du Cancer 2018 105 281–289. (10.1016/j.bulcan.2017.11.014) 29459090

[12] Smith LP Brubaker LW & Wolsky RJ. It does exist! Diagnosis and management of thyroid carcinomas originating in struma ovarii. Surgical Pathology Clinics 2023 16 75–86. (10.1016/j.path.2022.09.008) 36739168

